# Aescin and diosmin each alone or in low dose- combination ameliorate liver damage induced by carbon tetrachloride in rats

**DOI:** 10.1186/s13104-020-05094-2

**Published:** 2020-05-27

**Authors:** Sara Mahmmoud EL-Dakhly, Abeer Abdallah Ali Salama, Soha Osama Mahmoud Hassanin, Noha Nazeeh Yassen, Alaaeldin Ahmed Hamza, Amr Amin

**Affiliations:** 1 Faculty of Pharmacy, Modern University for Technology and Information, Cairo, Egypt; 2 Pharmacology Department, National Research Centre, Giza, Egypt; 3 Biochemistry Department, Modern University for Technology and Information, Cairo, Egypt; 4 Pathology Department, National Research Centre, Giza, Egypt; 5 Hormone Evaluation Department, National Organization for Drug Control and Research, Giza, Egypt; 6 Biology Department, UAE University, Al Ain, UAE; 7 Present Address: The University of Chicago, Chicago, United States

**Keywords:** Aescin, Diosmin, Carbon tetrachloride, Liver, Rats

## Abstract

**Objective:**

This study evaluated hepatoprotective effect of aescin (AES) and diosmin (DIO), individually or in low-dose combination in chemically induced liver injury in rats. Rats were divided into 6 groups; Group 1, control, Group 2, injected with a single dose of a mixture of corn oil and carbon tetrachloride (CCl_4_) to induce hepatic toxicity. Before CCl_4_ injection, Groups 3–6 were treated daily for 14 days with silymarin (SIL) (200 mg/kg), aescin (AES; 3.6 & 1.75 mg/kg), Diosmin (DIO; 100 & 50 mg/kg). Serum samples were analyzed for different liver function, oxidative stress and antioxidant markers. Moreover, inflammation and tissue damage were confirmed by histological staining of liver tissue sections.

**Results:**

Results indicated that CCl_4_ elevated serum levels of all assessed liver function markers and decreased levels of key antioxidants. Administration of AES and/or DIO significantly reversed all those CCl_4_-induced effects. Histopathological study showed disruption of the hepatic architecture, necrosis and inflammatory cells and depositions of glycogen and protein in the tissues of CCl_4_-treated group. Pretreatment with DIO and/or AES significantly improved histopathological structure of liver tissue. In conclusion, low-dose combination of AES and DIO exhibited significant and preferential hepatoprotective activity compared to individual treatment with AES or DIO.

## Introduction

Liver damage is viewed as a worldwide medical and economic burden that is often inclined by viral infection, poisonous synthetic concoctions, and hepatotoxic drugs [1]. The liver assumes an irreplaceable job in the digestion of xenobiotics and therapeutic agents being detoxified and excreted [2]. Chemically‐induced hepatic damage is mainly evoked by the metabolic change of chemical into free radicals, which can truly influence cell macromolecules [2]. Carbon tetrachloride (CCl_4_)—induced liver damage has been for quite some time utilized as hepatic damage model to evaluate the therapeutic potential of drugs. This model has also been used in mechanical liver injuries that are comparable to human liver disease both in morphology and the biochemical characteristics of cellular lesions [2]. This hepatotoxic agent induces hepatic necrosis through the generation of trichloromethyl free radical under the effect of cytochrome p-450 2E1 [3].

Previous investigations have reported that a wide spectrum of natural products can prevent or even treat different types of liver diseases [4–9]. β-Aescin (AES) is a triterpene saponin derived from the horse chestnut tree *Aesculus hippocastanum* L. (Hippocastanaceae). Traditionally, leaves, bark and seeds were used to treat arthritis, brain trauma, stroke, venous congestion and thrombophlebitis [10]. There are different formulations of AES in clinical applications, including oral tablets, injections, and topical gel. AES is widely used in the clinical therapy because of its anti-apoptotic, anti-edematous, anti-inflammatory, and antioxidant effects [11–13]. Moreover, some studies reported anti-inflammatory, anti-oxidative stress and protective effects of AES against liver damage induced in animals by endotoxin [14], methyl parathion [15] and CCl_4_ [16].

Diosmin (DIO) (3′5,7-trihydroxy-4′-methoxyflavone 7-rutinoside) is an unsaturated flavonoid glycoside, present in citrus fruits [17]. DIO formulations are used to treat chronic venous insufficiency, hemorrhoids, venous ulcers (especially of the lower limbs) and to prevent postoperative thromboembolism [18]. Previous investigations reported anti-inflammatory, anti-oxidative stress and protective effects of DIO against liver damage induced in animals by ethanol [19], methotrexate [20], iron [21] and by aflatoxin [22]. Moreover, DIO successfully relieved mitochondrial oxidative stress effect against isopropanol-induced myocardial injury in rats [23]. In that context, AES and DIO have received considerable attention to their tremendous potential for management of various forms of hepatopathy [14, 15, 19–22]. Assessing AES/DIO and their combination against induced-liver damage is the objective of this study.

## Main text

### Methods

#### Chemicals

All chemicals were provided as detailed in (Additional file 1).

#### Animals

Male Wistar rats, weighing 100–130 g, were provided by the animal house colony of the National Research Centre, Dokki, Giza, Egypt. Animal-related procedures were all approved and operated in accordance with the Ethics Committee of the National Research Centre and followed the recommendations of the National Institutes of Health Guide for Care and Use of Laboratory Animals.

#### Experimental design

Rats were randomized and divided into six groups (n = 6). Each was subjected to different treatment as detailed in (Additional file 1). Briefly, Control, received water (5 ml/kg b.wt.) for 14 days then vehicle (1 ml corn oil/kg b.wt.). CCl_4_ group received water (5 ml/kg b.wt) then CCl_4_ (50% CCl_4_/corn oil; 1 ml/kg body weight). Silymarin (SIL) + CCL_4_ group received SIL (200 mg/kg b.wt.) [24] and CCl_4_ (50% CCl_4_/corn oil; 1 ml/kg b.wt. i.p.). The AES + CCl_4_ group received AES (3.6 mg/kg b.wt.) [16] and CCl_4_ (50% CCl_4_/corn oil; 1 ml/kg b.wt. ip). DIO + CCl_4_ group received DIO (100 mg/kg) [25] and CCL_4_ (50% CCl_4_/corn oil; 1 ml/kg b.wt. ip). The ASE + DIO + CCl_4_ group received AES (1.75 mg/kg) and DIO (50 mg/kg) and then CCL_4_ (50% CCl_4_/corn oil; 1 ml/kg b.wt. ip).

#### Sample collection and preparation

Rats were anesthetized using diethyl ether then samples of blood were withdrawn from the retro-orbital plexus. Post blood collection, animals were euthanized by cervical dislocation under 3% sodium pentobarbital anesthesia and livers were swiftly dissected out, washed with cold normal saline and weighted. Blood samples were centrifuged at 3000 rpm g for 10 min and serum was used for further biochemical analyses.

#### Assessment of serum biochemical parameters

Kits used were purchased from sources detailed in (Additional file 1).

#### Histological and histochemical assessment studies

After blood collection, rats of each group were euthanized by cervical dislocation under 3% sodium pentobarbital anesthesia, and their livers were collected, dissected immediately after the sacrifice and were used for the histopathological analysis as described in [26, 27] and detailed in (Additional file 1).

#### Assessments of morphometric area percentage and optical density

This assessment was carried out on periodic acid Schiff (PAS) & Mercuric bromophenol blue stained slides. Assessed areas were analyzed as detailed in (Additional file 1).

#### Statistical analyses

Using statistical package for social science (SPSS), collected data were statistically analyzed as detailed in (Additional file 1).

### Results and discussion

#### AES, DIO and their combination attenuated liver lesions in CCl_4_-induced liver damage

**Fig. 1:**
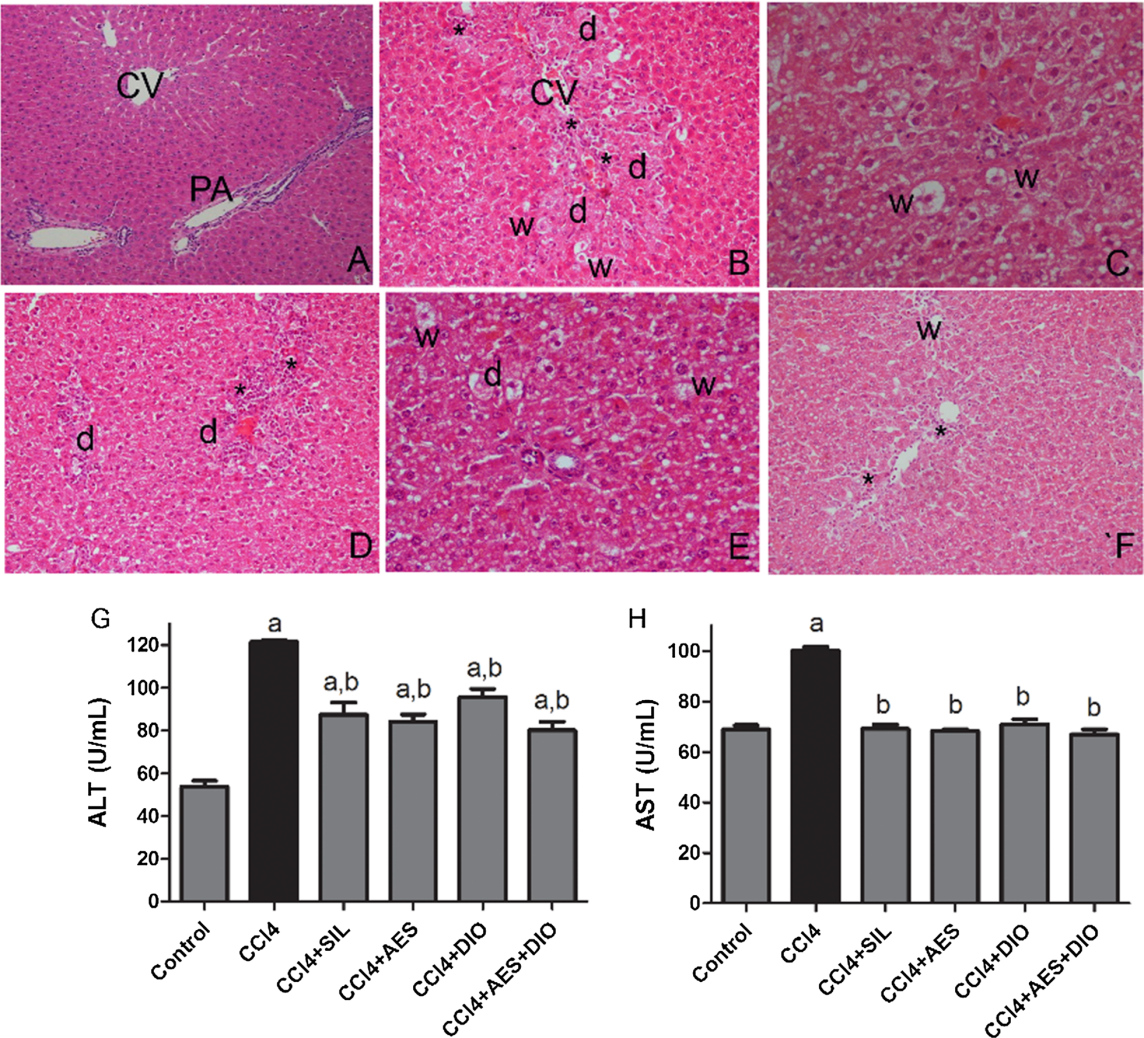
Effects of AES and DIO each alone or in low-dose combination on CCl_4_-induced liver damage in rats. (n = 6 per group). **A**–**F** Representative images of H&E-stained hepatic sections of the different groups (200 ×). C.V, (central vein), PA (portal area), Inflammatory cell infiltration (star), hepatic necrosis associated with degenerated nuclei (d) lipid droplets and vacuolization in hepatocytes (w). Serum markers of liver damage ALT (**G**), AST (**H**) activities. Data are presented as mean ± SEM. (n = 6/group). a P < 0.05 vs. control group. b P < 0.05 vs. CCL_4_ group

Normal liver sections “control group” showed typical hepatic architecture with the central vein centrally located and normally thickened hepatic cords radiating with well-formed hepatocytes with centrally located nuclei and intact cell membrane (Fig. 1A). CCl_4_-induced group showed hepatocytes degenerative changes including pyknotic nuclei. In addition, central veins were massively enlarged and clogged with ductular cells hyperplasia, scattered inflammatory cells which were concentrated around hepatic vessels (Fig. 1B). Compared to the SIL + CCl_4_ group (Fig. 3C) and to the protected groups; treated with ASE (Fig. 3D), DIO (Fig. 1E), animals intoxicated with CCl_4_ and treated with both AES and DIO at low dose combination showed marked degree of improvement of hepatic tissue as the hepatic tissue restored its normal structure and architecture with centrally located central vein and intact hepatocytes (Fig. 1F). Currently, CCl_4-_induced liver injury is a model that is widely employed to screen for anti-hepatotoxic/hepatoprotective drugs. It is also a useful tool to study different liver illnesses, such as fatty liver, fibrosis, and cirrhosis [2, 28]. The magnitude of CCl_4_ hepatic impairment is evaluated by the enhanced serum level of cytoplasmic enzymes and by histopathologic changes in liver [29, 30].

Compared to control, CCl_4_-induced group had significantly higher levels of serum alanine aminotransferase (ALT) (130%), and aspartate aminotransferase (AST) (40%) (Fig. 1G, H). Levels of ALT and AST were significantly (P ≤ 0.05) improved upon treatment with SIL, AES and DIO. Pretreatment with SIL, AES, DIO and their combination reduced the elevated serum ALT by 28%, 34%, 31% and 21%, respectively, and AST levels by 31%, 33%, 29% and 32%, respectively, compared to animals in CCl_4_ group (Fig. 1G, H). Upregulation of ALT and AST have been ascribed to hepatotoxicity as they are released into blood serum upon hepatocyte degeneration [31]. The current study showed quite a restoration of almost normal levels especially when combined treatment was applied.

#### Glycogen and proteins distribution

**Fig. 2:**
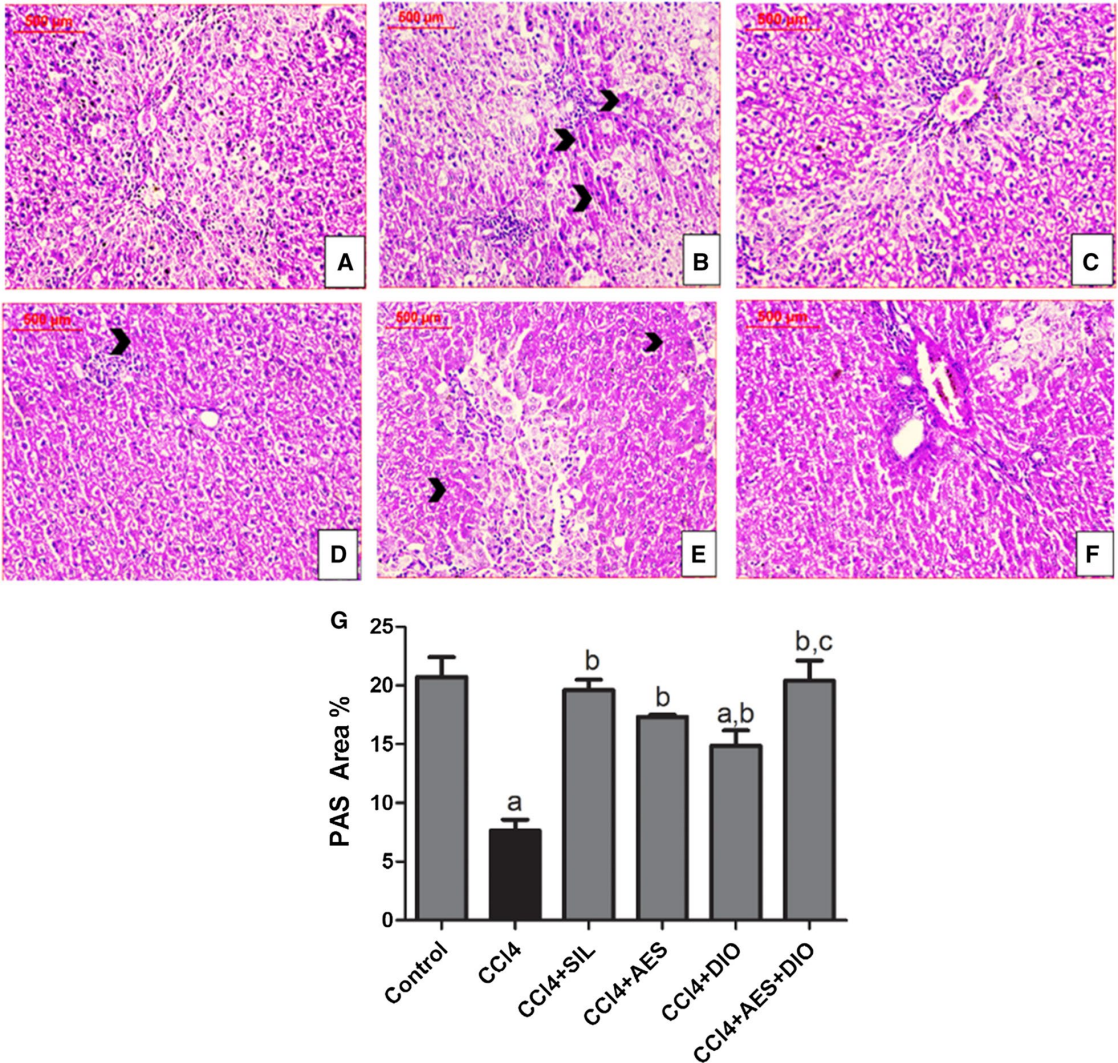
Effects of AES and DIO each alone or in low-dose combination on the distribution of glycogen contents in liver tissue stained by PAS reagent in the CCl_4_-induced liver rat model. **A**–**F** Representative images of PAS staining (red area staining, arrow heads). 200 × magnification, scale bar equals 500 µm. **G** PAS -positive area expression as a percentage of the total area across10 different fields for each rat section using Leica Qwin 500 Image Analyzer. Data are presented as mean ± SEM (n = 6/group). a P < 0.05 vs. control group. b P < 0.05 vs. CCL_4_ group, c P < 0.05 vs SIL group

**Fig. 3:**
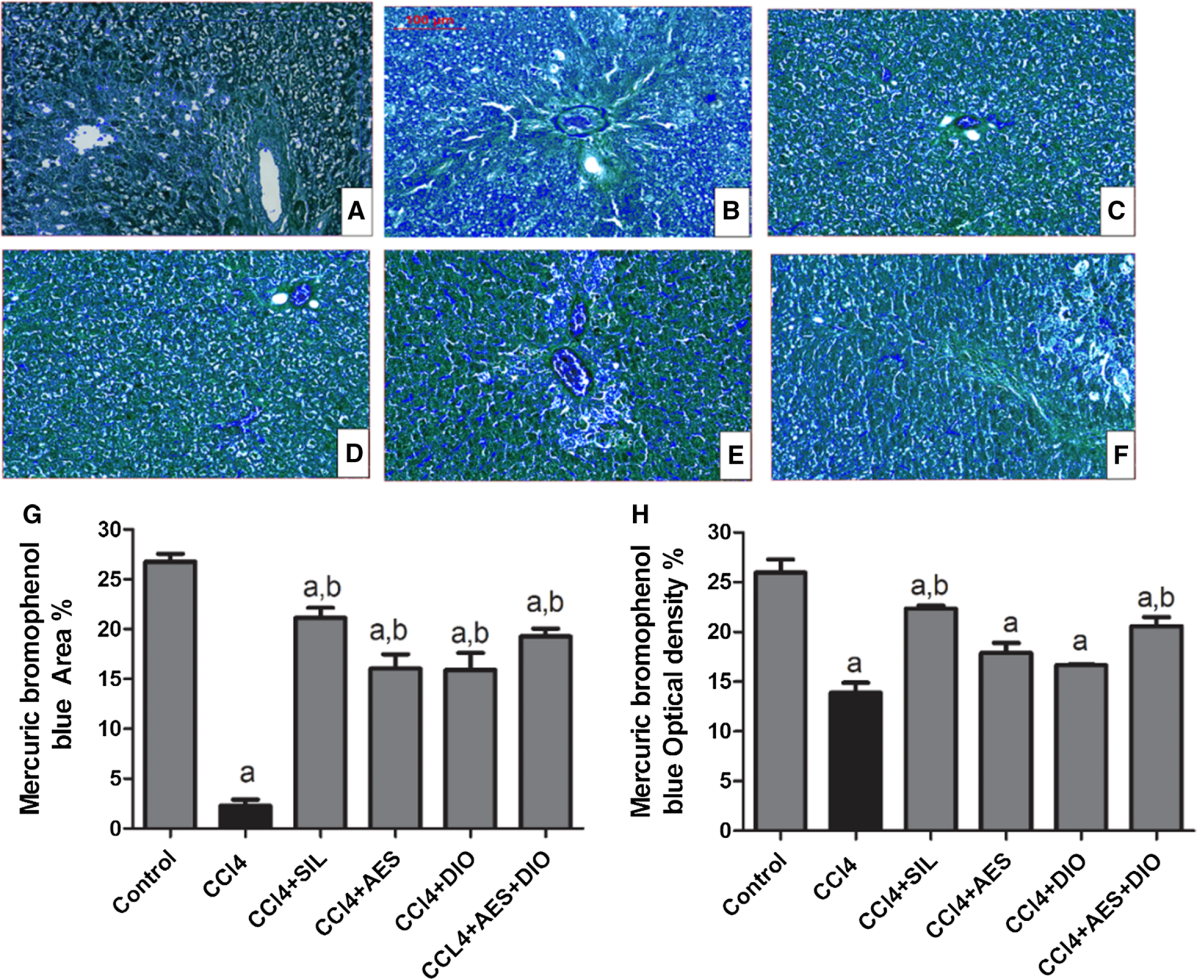
Effects of AES and DIO each alone or in low-dose combination on the distribution of protein contents in liver tissue stained by Mercuric bromophenol blue reagent in the CCl_4_-induced liver rat model. **A**–**F** Representative images of mercuric bromophenol blue staining (blue area), 200 × magnification, scale bar equals 500 µm. Mercuric bromophenol blue-positive area expression  % (**G**) and optical density  % (**H**) across10 different fields for each rat section using Leica Qwin 500 Image Analyzer. Data are presented as mean ± SEM (n = 6/group). a P < 0.05 vs. control group. b P < 0.05 vs. CCl_4_ group

Figure 2 shows the distribution of glycogen contents in normal liver tissue as reflected by PAS positivity area (Fig. 3A; 20.74 ± 1.65). CCl_4_ group showed severe depletion of glycogen 6.63 ± 1.6 with a reduction percent of 82.45% from the control group.

Groups treated with DIO, AES and both showed varying improvements in the glycogen content as reflected by the PAS area  % of 15.84 ± 1.23, 16.54 ± 0.79 and 22.56 ± 1.36 respectively. Hepatotoxicity is always correlated with disruption in the synthesis and metabolism of glycogen and proteins. From the present findings, both protein and glycogen were reduced in CCl_4_ group. Poisonous effect on liver may mainly contribute to such reduction through the drug-induced necrosis of the plasma membrane, or via depleting energy sources needed for synthesizing protein and other metabolic events through obstructing the oxidative phosphorylation activity as documented elsewhere [32]. All tested preventive treatments successfully reversed such effect.

The control group showed the highest protein content (as reflected by Mercuric bromophenol blue positively stained area of the hepatocytes and its optical density of 26.75 ± 0.812 and 25.76 ± 1.66; Fig. 3). CCl_4_-intoxicated group showed the lowest comparable values of 2.29 ± 0.61 and 13.9 ± 0.98. Protected groups treated by AES and DIO showed varying degrees of protein content improvements; 16.037 ± 1.45 and 15.913 ± 1.7 and optical density of 17.88 ± 1.01 and 16.66 ± 0.08 respectively. The combined administration of AES and DIO at low dose, strengthened the stain reaction and showed marked improvement in the protein content of hepatocytes of 19.27 ± 0.8 and optical density of 20.59 ± 0.9. The present results of pre-administration of AES improved liver histology and function and significantly enhanced total hepatic glycogen and protein content. These results confirmed the hepatoprotective effect of AES as previously reported against liver damage induced by methyl parathion [15] and CCl_4_ [16, 22] in rats. Although treatment with DIO do positively contribute to liver histology and function, the protective effect of DIO was less evident than that of AES. These hepatoprotective effects of DIO coincide with those of [15, 19, 20]. Interestingly, livers of rats in CCl_4_ group treated with both AES and DIO showed minimal inflammation, eosinophilic staining and degenerations with significant enhancements in overall glycogen and protein contents in liver.

#### AES, DIO and their combination ameliorated oxidative stress and inflammation in CCl_4_-induced liver damage in rats

The administration of SIL, AES, DIO and AES/DIO combination significantly attenuated the increment in serum MDA and nitric oxide (NO) levels and partly attenuated the decrement in glutathione (GSH), Catalase and protein kinase C (PKC). Compared to SIL, AES, DIO and AES/DIO groups, the treatment with combined AES and DIO showed the best effects against all examined oxidative stress markers (Additional file 2). Abundant evidence suggests that reactive oxygen species (ROS) and other free radicals are induced following hepatic insults with drugs like CCl_4_. The enzymatic/non-enzymatic defense system is the natural protec-tor against free radicals accumulation [28, 30]. The accumulation of free radicals can cause decreased Catalase and GSH levels and a decline in capacity of scavenging free radicals. Consisting with previous report [30, 33], liver damage shown in CCl_4_-treated group was associated with enhanced MDA and NO as well as depletion of GSH and Catalase. Oxidative stress is known to cause activation of several stress kinases such as PKC and leads to exacerbation of cell toxicity [34]. The elevation of PKC in the present work was in line with the results of the previous studies which suggested that the hepatic oxidative stress and injury induced by bezafibrate [34] and acetaminophen [35] and CCl_4_ [36] was mediated by elevation of PKC. PKC induces upregulation of tumor necrosis factor- α (TNF-α), interleukin-6 (IL-6), and ROS, a major contributor to CCl_4_-induced liver injury [36]. Here, and in agreement with results of [16], AES ameliorated CCl_4_-induced hepatotoxicity by reducing MDA and NO and increasing GSH activity. Similarly, in this study, DIO mitigated CCl_4_-induced oxidative and nitrative stresses confirming previous studies that showed that DIO alleviates the oxidative stress caused by aflatoxin [22], methotrexate [20] by increasing GSH levels, lowering NO levels, and enhancing the activity of antioxidant enzymes. Most interestingly, much improvement in oxidative stress and antioxidants status was observed here when AES was combined with DIO.

## Limitations

This study was primarily focused on the assessment of tested extracts in induced liver fibrosis in animal model, more investigations are required for more understanding of the molecular mechanisms by which AES and DIO each alone or in low-dose combination might exert their beneficial hepatoprotective effect such as their effects on the inhibition of cytochrome CYP2E1 activity.

## Supplementary information

**Additional file 1.** Detailed methods.

**Additional file 2.** Effects of AES and DIO each alone or in low-dose combination on oxidative stress and inflammatory markers in rats. Data are presented as mean ± SEM (n = 6/group). a P < 0.05 vs. control group. b P < 0.05 vs. CCl_4_ group, c P < 0.05 vs SIL group, d P < 0.05 vs AES group. e P < 0.05 vs DIO group, f P < 0.05 vs AES +DIO.

## Data Availability

All data supporting the conclusions of this article are included within the article and all datasets supporting our findings are available.

## Abbreviations

AES: Aescin
DIO: Diosmin
CCl_4_: Carbon tetrachloride
SIL: Silymarin
PAS: Periodic acid Schiff
SPSS: Statistical package for social science
ALT: Alanine aminotransferase
AST: Aspartate aminotransferase
MDA: Malondialdehyde
NO: Nitric oxide
GSH: Glutathione
PKC: Protein kinase C
TNF-α: Tumor necrosis factor α
IL-6: Interleukin-6
ROS: Reactive oxygen species
