# Supplementary material for: Aescin and diosmin each alone or in low dose- combination ameliorate liver damage induced by carbon tetrachloride in rats
Source: BMC Res Notes. 2020 May 27;13:259. doi: 10.1186/s13104-020-05094-2 (PMC7251915; doi:10.1186/s13104-020-05094-2)
Supplement: Supplementary file 1 — Additional file 1. Detailed methods. [file 13104_2020_5094_MOESM1_ESM.docx]

**File name: Additional file 1_Detailed methods**

***Chemicals***

CCl_4_ was purchased from El-Gomhouria Company for drug and chemicals, Egypt. SIL, AES and DIO were obtained from Sigma-Aldrich, USA. All other chemicals, used throughout the experiment, were of the highest analytical grade available.

***Animals***

Male Wistar rats, weighing 100-130g, were used throughout the experiments. Rats were obtained from the animal house colony of the National Research Centre, Dokki, Giza, Egypt. The animals were housed 3 per cage in standard metal cages with autoclaved bedding in a pathogen-free air-conditioned room at 22± 3 ^o^C, 55± 5% humidity and provided with standard laboratory diet and water *ad libitum*. Throughout the study automatic timers maintained 12 hours light cycle; lights on at 6 a.m. and off at 6 p.m. Animals were left to acclimatize for 7 days before the start of the study. Experiments were performed between 9:00 AM and 15:00 PM. Animals were monitored daily by husbandry staff. All animal-related procedures were performed and approved in accordance with the Ethics Committee of the National Research Centre and followed the recommendations of National Institute of Health Guide for Care & Use of Laboratory Animals.

***Experimental design***

The rats were randomly divided into six groups (n = 6) and were subjected to the following treatments: The first group, “Control”, received a daily oral dose of water (5ml /kg b.wt.) for 14 days then a single dose of corn oil (1ml corn oil / kg b.wt.) was injected intraperitoneally (i.p.) into each animal of the this group. Animals of the second group, “CCl_4_” received a daily oral dose of water (5ml /kg b.wt) for 14 days and then a single dose of CCl_4_ (50 % CCl_4_ / corn oil; 1ml/kg body weight). The third group, “SIL+CCL_4_” was a reference group, where animals received a daily oral dose of SIL (200 mg / kg b.wt.) [[24](#_ENREF_18)] for 14 days and a single dose of CCl_4_ (50% CCl_4_ / corn oil; 1ml/kg b.wt. i.p.). Thanks to its hepatoprotective and anti-fibrotic properties, SIL is the most clinically utilized drug by patients and is used here as reference control [[24](#_ENREF_18)]. The fourth group, “AES+ CCl_4_“ has received a daily oral dose of AES (3.6 mg / kg b.wt.) [[16](#_ENREF_10)] for 14 days and a single dose of CCl_4_ (50% CCl_4_ / corn oil; 1ml/kg b.wt. ip). The fifth group, “DIO+ CCl_4_“ received a daily oral dose of DIO (100 mg/kg) [[25](#_ENREF_19)] for 14 days and a single dose of CCL4 (50% CCl_4_ / corn oil; 1ml/kg b.wt. ip). The sixth group, “ASE+DIO+ CCl_4_“ received a daily oral dose of AES (1.75 mg/kg) and DIO (50 mg/kg) for 14 days and then a single dose of CCL4 (50% CCl_4_ / corn oil; 1ml/kg b.wt. ip). Selected treatment’s doses were determined based on dose-response pilot studies (not shown here). The time interval between CCl_4_ and each of ASE and DIO were 5 hours to avoid the disturbance of absorption of each agent.

***Sample collection and preparation***

24h post CCl_4_ injection, all rats were anesthetized with diethyl ether and blood samples were collected from the retro-orbital plexus. After the blood withdrawal, the animals were euthanized by cervical dislocation under diethyl ether anesthesia and the liver was quickly taken out and weighted after washed with cold normal saline. Serum was separated by centrifugation at 3000 rpm g for 10 min and used for biochemical serum analysis.

***Assessment of serum biochemical parameters***

Kits used to measure serum aspartate transaminase (AST), alanine transaminase (ALT), nitric oxide (NO; measured as nitrate and nitrite by using Griess Reagents), malondialdehyde (MDA), glutathione (GSH) and catalase were all purchased from Biodiagnostic, Inc., (Egypt). Protein kinase C (PKC) enzyme linked immunoassay (ELISA) technique standard kit was obtained from Glory Science Co., Ltd, USA.

***Histological and histochemical assessment studies***

After blood collection, rats of each group were sacrificed under diethyl ether anesthesia, and their livers were collected, dissected immediately after the sacrifice and were used for the histopathological analysis. For that, tissues’ specimens were fixed in 10 % neutral-buffered formalin saline for 72 hours at least. All the specimens were washed in tap water for 30 min., dehydrated in ascending grades of alcohol, cleared in xylene and finally embedded in paraffin. Serial sections of 5μm thick were cut and stained with haematoxylin and eosin (H & E) for histopathological investigation. Glycogen deposition was detected by periodic acid Schiff (PAS) method [26] and proteins deposition was detected by Mercuric bromophenol blue method [[27](#_ENREF_21)].

The mercuric bromophenol blue is a method for protein staining in fixed sections where slides were stained with 1% HgCl2 and 0.01% bromophenol blue in 2% aqueous acetic acid for 15 min at room temperature. Slides were then rinsed for 20 min in 2 changes of 0.5% aqueous acetic acid, washed for 3 min in neutral aqueous solution for color differentiation and were finally embedded in absolute ethanol with agitation before transferring to xylene.

***Assessments of morphometric area percentage and optical density***

This assessment was carried out on PAS & Mercuric bromophenol blue stained slides. Assessed area was measured using the Leica Qwin 500 Image Analyzer (LEICA Imaging Systems Ltd, Cambridge, England) at the Pathology Department, National Research Centre, Cairo, Egypt. This area is determined as an area per field in micrometer square, area fraction and area percentage by using the interactive software of the system. The results appear automatically on the monitor in the form of a table with the total, mean, standard deviation, standard error, the minimum area and the maximum area measured. The area of glycogen and protein measured in 10 fields randomly chosen, in each slide. Mean optical density (MOD) measurement of Mercuric bromophenol blue histochemical stain was performed using the Leica Qwin 500 Image Analyzer (LEICA Imaging Systems Ltd, Cambridge, England). All slides were examined at high power magnifications 200×, and then MOD was measured in 10 fields randomly chosen. The final MOD value was calculated according to the software ranges from 0 (zero = most light stain, lowest expression), to 256 (256 = most dense stain, highest expression) with illustrating histograms describe the number of cells taking such MOD value.

***Statistical analyses***

All data were statistically analyzed by SPSS (version 20) statistical program (SPSS Inc., Chicago, IL, USA). The data were expressed as means ± SEM. Statistical significance between treatment groups was carry out by using one-way analysis of variance (ANOVA) followed by Tukey post-hoc test for multiple comparisons with P < 0.05 being considered as statistically significant. Figures were plotted using GraphPad Prism program (version 5) (San Diego, California, USA).
