# Supplementary material for: Aescin and diosmin each alone or in low dose- combination ameliorate liver damage induced by carbon tetrachloride in rats
Source: BMC Res Notes. 2020 May 27;13:259. doi: 10.1186/s13104-020-05094-2 (PMC7251915; doi:10.1186/s13104-020-05094-2)
Supplement: Supplementary file 2 — Additional file 2. Effects of AES and DIO each alone or in low-dose combination on oxidative stress and inflammatory markers in rats. Data are presented as mean ± SEM (n = 6/group). a P < 0.05 vs. control group. b P < 0.05 vs. CCl4 group, c P < 0.05 vs SIL group, d P < 0.05 vs AES group. e P < 0.05 vs DIO group, f P < 0.05 vs AES +DIO. [file 13104_2020_5094_MOESM2_ESM.docx]

**File name: Additional file 2**

**Title of data:** Effects of AES and DIO each alone or in low-dose combination on oxidative stress and inflammatory markers in rats. Data are presented as means ± SEM (n = 6 / group). a P<0.05 vs. control group. b P<0.05 vs. CCl4 group, c P<0.05 vs SIL group, d P<0.05 vs AES group. e P<0.05 vs DIO group, f P<0.05 vs AES +DIO.

**Description of data:** Effects of AES and DIO each alone or in low-dose combination on oxidative stress and inflammatory markers in rats.

**
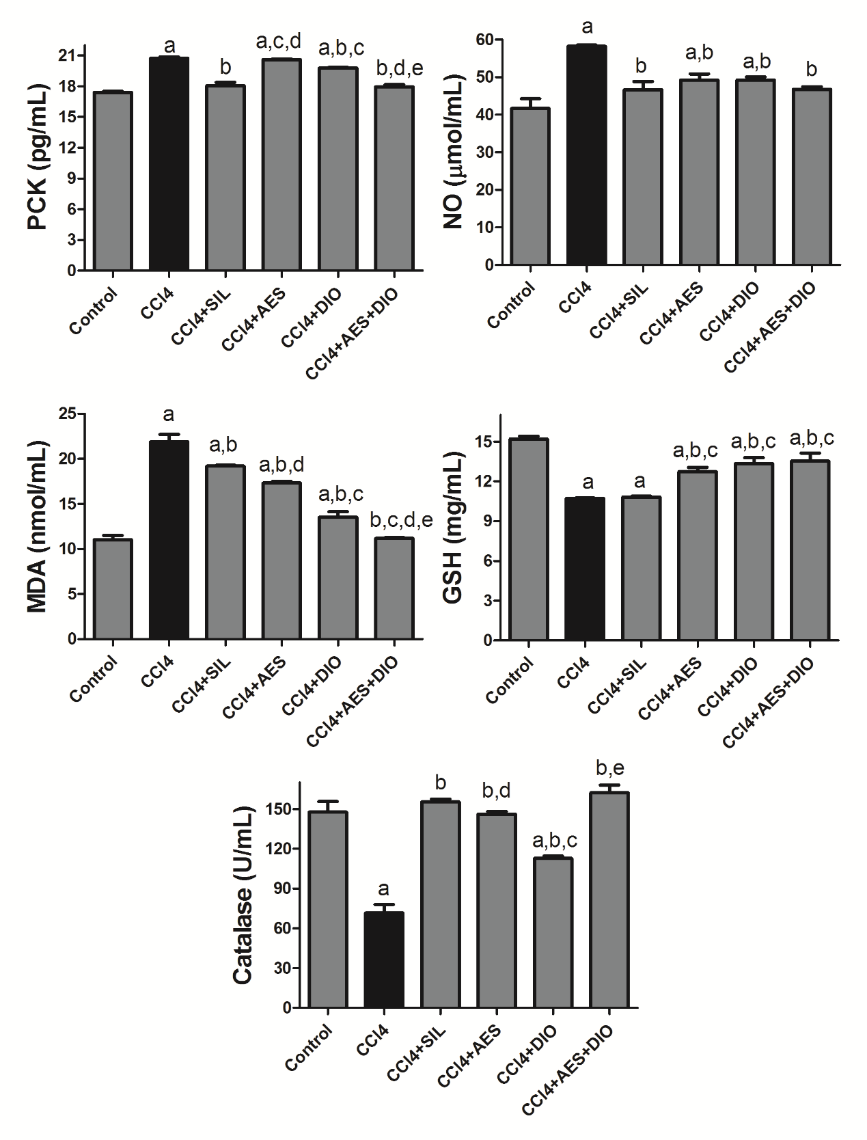
**

**Additional file 2**. Effects of AES and DIO each alone or in low-dose combination on oxidative stress and inflammatory markers in rats. Data are presented as means ± SEM (n = 6 / group). a P<0.05 vs. control group. b P<0.05 vs. CCl_4_ group, c P<0.05 vs SIL group, d P<0.05 vs AES group. e P<0.05 vs DIO group, f P<0.05 vs AES +DIO.
